# miRNAs Epigenetic Tuning of Wall Remodeling in the Early Phase after Myocardial Infarction: A Novel Epidrug Approach

**DOI:** 10.3390/ijms241713268

**Published:** 2023-08-26

**Authors:** Francesca Salvatori, Elisabetta D’Aversa, Maria Luisa Serino, Ajay Vikram Singh, Paola Secchiero, Giorgio Zauli, Veronica Tisato, Donato Gemmati

**Affiliations:** 1 Department of Translational Medicine, University of Ferrara, 44121 Ferrara, Italy; slvfnc@unife.it (F.S.);; 2 Centre Haemostasis & Thrombosis, University of Ferrara, 44121 Ferrara, Italy; 3 Department of Chemical and Product Safety, German Federal Institute for Risk Assessment (BfR), 10589 Berlin, Germany; 4 Department of Environmental Science and Prevention, University of Ferrara, 44121 Ferrara, Italy; 5 LTTA Centre, University of Ferrara, 44121 Ferrara, Italy; 6 University Centre for Studies on Gender Medicine, University of Ferrara, 44121 Ferrara, Italy

**Keywords:** myocardial infarction, epigenetic tuning, miRNAs, sex-gap, epidrugs

## Abstract

Myocardial infarction (MI) is one of the leading causes of death in Western countries. An early diagnosis decreases subsequent severe complications such as wall remodeling or heart failure and improves treatments and interventions. Novel therapeutic targets have been recognized and, together with the development of direct and indirect epidrugs, the role of non-coding RNAs (ncRNAs) yields great expectancy. ncRNAs are a group of RNAs not translated into a product and, among them, microRNAs (miRNAs) are the most investigated subgroup since they are involved in several pathological processes related to MI and post-MI phases such as inflammation, apoptosis, angiogenesis, and fibrosis. These processes and pathways are finely tuned by miRNAs via complex mechanisms. We are at the beginning of the investigation and the main paths are still underexplored. In this review, we provide a comprehensive discussion of the recent findings on epigenetic changes involved in the first phases after MI as well as on the role of the several miRNAs. We focused on miRNAs function and on their relationship with key molecules and cells involved in healing processes after an ischemic accident, while also giving insight into the discrepancy between males and females in the prognosis of cardiovascular diseases.

## 1. Introduction

**Figure 1:**
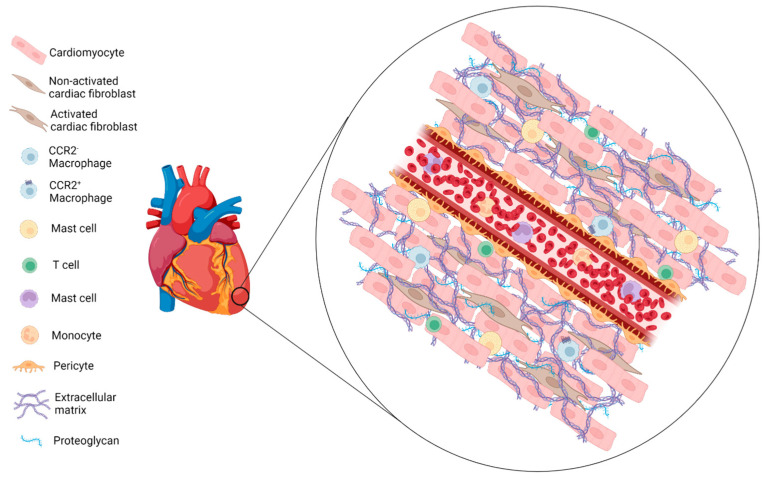
**Cardiac cell types and microenvironment.** Schematic representation of myocardial tissue architecture, which is mainly supported by ECM proteins. The most relevant cell types present in the adult steady-state heart are shown. Created with BioRender.com.

The heart is a highly specialized organ, whose continuous contraction allows perfusion, oxygenation and, therefore, the survival of cells and tissues. The cardiomyocytes (CMs) responsible for contraction are only one of the numerous cell types present in the heart and involved in its homeostasis. In fact, they do not act alone but work as part of a complex network made up of specialized cells, including those involved in tissue vascularization, the remodeling of the interstitial space, the regulation of contractions and in the innate and adaptive immune response [1] (Figure 1).

Although CMs represent the functional “mass” of the heart, fibroblasts are present in equivalent numbers. Cardiac fibroblasts (CFs) are cells of mesenchymal origin capable of secreting components of the extracellular matrix (ECM), but in the heart, they are also involved in regulating cardiac form and function, mainly through paracrine effects via the secretion of growth factors and cytokines. Fibroblasts communicate with myocytes and endothelial cells (ECs), allowing for the regulation of electrophysiological properties and increased blood vessel formation [1,2]. Another important cellular component of the heart is represented by resident immune cells, which have an important role in cardiac homeostasis, immunological and repair processes [3]. Apart from a small population of T cells and neutrophils, most of the resident immune cells are made up of macrophages and mast cells (MCs) [1,4]. The latter are located mainly in the pericardial adipose tissue and appear to be of the MC_TC_ subtype containing tryptase, chymase, cathepsin G, and carboxypeptidase and are usually found in connective tissues. Cardiac MCs also contain tumor necrosis factor-alpha (TNF-α) and transforming growth factor-beta (TGF-β), involved in myocardial remodeling [5,6,7,8].

The most common resident immune cells in the heart are macrophages. They can be classified into two subgroups based on the expression of C–C motif chemokine receptor 2 (CCR2+ and CCR2−), which binds to monocyte chemoattractant protein 1 (CCL2), which allows monocyte infiltration during inflammation. The presence of this receptor also allows us to discriminate between the macrophages that derive from yolk-sac precursors (CCR2−) and those that develop from circulating monocytes (CCR2−). The CCR2− and CCR2+ macrophages show different functions in the healing heart, which will be discussed later [9,10]. Macrophages of embryonic origin proliferate autonomously in the heart, keeping their number constant, although the proliferation rate decreases over time [11,12]. Conversely, CCR2+ macrophages are regularly replaced by bone marrow or extramedullary tissues derived monocytes [13]. CCR2− cardiac-resident macrophages can engulf dying cells, cellular debris, or pathogens [14], and they play a key role in the proper development of the heart’s vasculature [15], angiogenesis [16], lymphangiogenesis and maturation of lymphatic vessels [17]. Moreover, being widely present in the atrioventricular as well as in the myocardium, they appear to improve cardiac conductivity, acting as a “bridge” between CMs that are not in direct contact via gap junctions (mainly gap junction alpha-1 protein (Cx43) protein) [4,9,18].

### Epigenetic Regulation

Various features of cardiac cells are regulated by epigenetic factors. It has been long established that CMs, which are very active in replication during the embryonic-fetal period, drastically reduce their proliferative capacity after birth, leading to the maximum number of cells being reached one month after birth. Since then, the increase in heart size is mainly due to cell hypertrophy, although evidence highlights a minimal replicative capacity of adult CMs, though not sufficient enough to regenerate heart tissue following injury [19].

DNA methylation is the main epigenetic form of gene regulation, and it mainly occurs at the level of the CpG islands in the promoter regions, so gene expression in adult CMs is partially regulated by the methylation of CpG islands. Of note, Gilsbach and colleagues identified 79,655 differently methylated regions (DMRs) by comparing adult CMs with embryonic stem cells (ESCs) [20]. Of these, 90% are hypermethylated while only 10% are hypomethylated, where demethylation directly correlates with active histone marks and increased expression of cardiomyocyte genes [20]. This correlation is not always maintained, since not all demethylated genes in CMs with respect to ESCs are effectively expressed. Many of these methylation changes are associated with trimethylated histone H3 at amino acid K27 (H3K27me3), which is in turn associated with polycomb-mediated gene repression and thus with blocking of the expression [20]. 

Another gene regulatory mechanism highly involved in CMs biology is histone acetylation. Acetylation of lysines reduces the charge of histones, resulting in the loss of their electrostatic attraction to DNA. This allows for the opening of the chromatin and, therefore, the activation of gene transcription. Chromatin hyperacetylation is present in embryonic cells (H3K9/14, H3K18, and H3K27), concomitant with high expression of p300 acetyltransferase, which decreases rapidly after birth, resulting in hypoacetylation of histones [21,22]. Accordingly, p300 acetyltransferase has been considered a putative epidrug target to contrast cellular damage related to aging and cardiovascular (CV) disease [23]. Epigenetic-based therapy is capturing growing interest due to the benefits of direct (e.g., Apabetalone) or indirect epidrugs such as statins, metformin, SGLT2i, and W-3 via epigenome modifications. On the other hand, also already available drugs have potential indirect epigenetic effects (e.g., metformin, statins, sodium-glucose transporter inhibitors 2, phytochemicals and nutritive vitamins/supplements). This paves the way for the development of novel therapeutics for the treatment of major adverse CV events [24,25].

Histone deacetylases (HDACs) are also involved in the regulation of CMs gene expression and proliferation. In particular, class I HDACs seem to be involved in the suppression of proliferation, such as HDAC2, which interacts with the cardiac negative regulator HOPX (homeodomain-only), reducing acetylation of GATA4 with consequent suppression of CMs cycling [26]. In contrast, class II HDACs, such as HDAC5 and HDAC9, appear to be involved in the stimulation of CMs proliferation. The activities of HDAC5 and HDAC9 have been shown to be redundant, which is why the loss of one of their genes does not drastically alter the effects on the heart [27].

**Figure 2:**
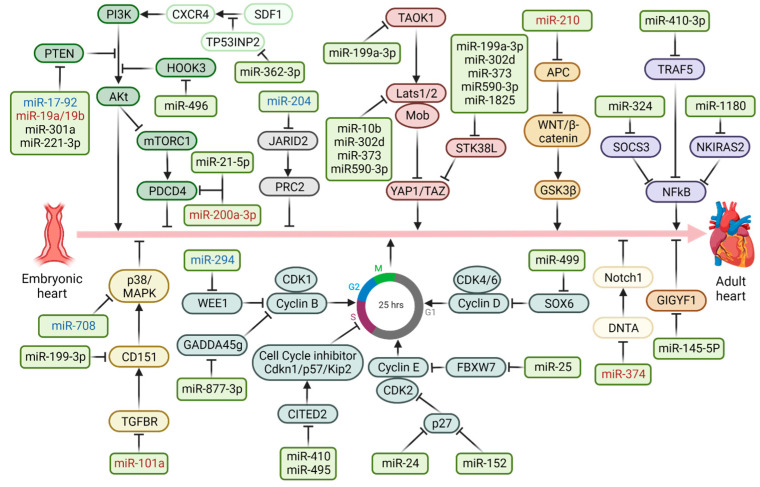
**Pro-proliferative miRNAs regulating heart development.** Schematic representation of key signaling pathways involved in CMs regulation. Relevant miRNAs involved in cell proliferation are shown. Black: embryonic/neonatal; Blue: neonatal adult; Red: adult. Created with BioRender.com.

**Figure 3:**
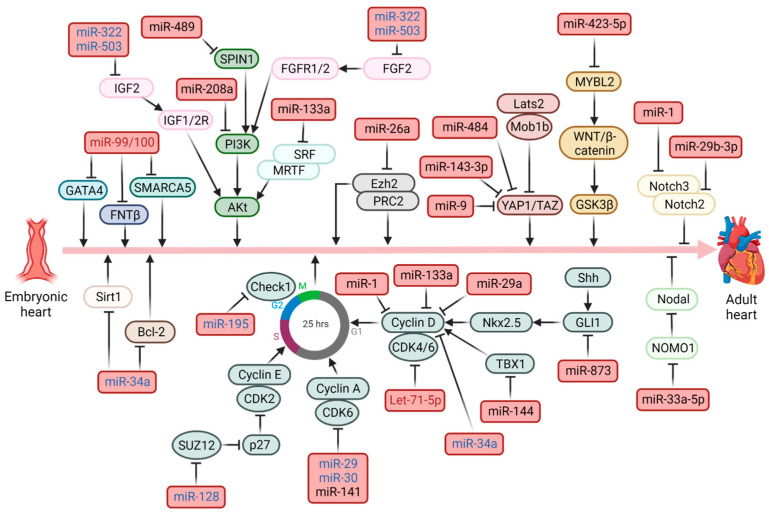
**Anti-proliferative miRNAs regulating heart development.** Schematic representation of the key signaling pathways involved in the regulation of CMs. Relevant miRNAs involved in the anti-proliferative, pro-apoptotic effect are shown. Black: embryonic/neonatal; Blue: neonatal adult; Red: adult. Created with BioRender.com.

Another important aspect of gene regulation in CMs is related to the effects of non-coding RNAs (ncRNAs), long non-coding RNAs (lncRNAs) and microRNAs (miRNAs). Among these, miRNAs are certainly the best characterized and studied non-coding RNAs. They consist of 18–22 nucleotides and allow for the regulation of gene expression by binding to the 3′ untranslated region (3′UTR) of the transcript, stimulating its degradation or blocking its protein translation [28]. The miRNAs involved in the regulation of CMs can be divided into those that stimulate the proliferation and/or hypertrophy, defined as “pro-proliferative” (Figure 2), and those that instead reduce the proliferative capacity or increase apoptosis, defined as “pro-apoptotic” (Figure 3). In both cases, miRNAs show multiple ways of action directly on cell cycle regulatory proteins, such as the pro-proliferative miR-499 which blocks the expression of SOX6, activating accordingly the cyclin D1 promoter [29]. Another example is the pro-apoptotic miR-195 which inhibits the expression of checkpoint kinase 1 (Check1) [30,31], which regulates G1/S transition, S phase, mitotic entry and mitosis [32]. miRNAs also regulate CMs proliferation through several signaling pathways, some of which are targeted by both pro-proliferative and pro-apoptotic miRNAs. This is the case of the Akt pathway, whose kinase activity can be increased by inhibitors of the expression of the PTEN tumor suppressor, such as miR17-92, miR-19a/b or miR-221-3p [33,34,35] or silenced by miR-208a and miR-489, which block phosphoinositide 3-kinase (PI3K) and spindlin-1 (SPIN1), respectively [36,37]. Conversely, other pathways appear to be regulated only by pro-proliferative or pro-apoptotic miRNAs, such as the NF-kB pathway which can be activated by blockade of the suppressor of cytokine signaling 3 (SOCS3) and the NF-kB inhibitor interacting Ras-like 2 (NKIRAS2) by miR-324 [38] and miR-1180 [39], respectively. However, the Nodal signaling pathway, an important signal transduction pathway active during embryonic heart development [40], is suppressed by miR-33a-5p which targets nodal modulator 1 (NOMO1) [41].

Summarizing, Table 1 and Table 2 show the main pro-proliferative and pro-apoptotic miRNAs studied so far, most of which are mainly found in the embryonic-neonatal stage, while others have also been identified in the adult stage. Certainly, the various aspects of the epigenetic regulation of CMs proliferation are not totally separate from each other. For example, both the pro-proliferative miR-204 [42] and the pro-apoptotic miR-26a [43] target the polycomb repressive complex with histone methyltransferase activity, involved in the silencing of genes in hypomethylated regions of CMs genome [20], while HDAC3 promotes myocardial growth by stimulating fibroblast growth factor-9 (FGF9) and insulin-like growth factor-2 (IGF2) through repressing the pro-apoptotic miR-322 and miR-503 [44].

## 2. Myocardial Infarction

**Figure 4:**
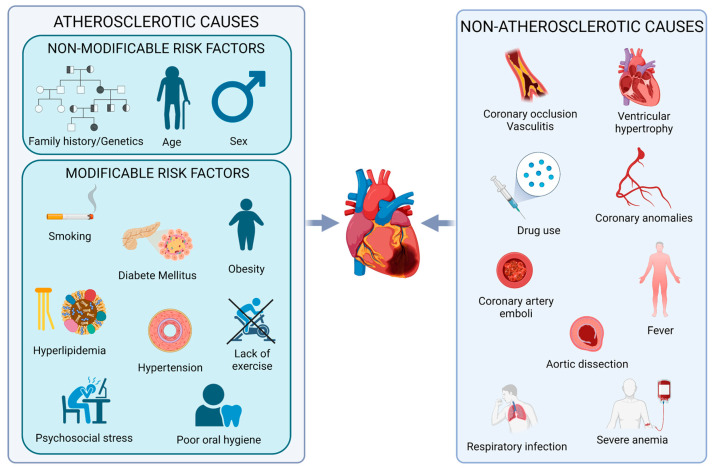
**MI etiopathogenesis.** Schematic representation of the main risk factors for MI divided into atherosclerotic dependent (**left** panel) and non-atherosclerotic causes (**right** panel). Created with BioRender.com.

Myocardial infarction (MI) is the result of acute or chronic ischemia, due to a significant reduction of blood flow responsible for cutting in oxygen and glucose supply, leading to myocardial injury up to tissue death [81]. Although it may have a complex multifactorial origin, atherosclerosis represents the primary etiopathological risk factor. Atherosclerosis in turn can be caused by modifiable risk factors, such as hyperlipidemia, hypertension, diabetes mellitus, obesity, smoking, psychosocial stress, lack of exercise and a sedentary lifestyle, and non-modifiable risk factors such as genetic predisposition together with age progression, male gender and positive family history [82,83,84,85,86] (Figure 4).

MI of atherosclerosis origin arises from intracoronary occlusive thrombosis superimposed on a pre-existent atherosclerotic plaque. The thrombus is usually produced following fissuring or ulceration of the plaque with consequent exposure to the bloodstream of its core or tissue factor and subsequent activation of the thrombogenic response [87,88]. In detail, specific actions of inherited risk/protective factors have been reported by investigating selective haplotypes in coagulation factors genes such as *F13A1*, *F13B*, *F7* and *F5*, resulting in modified levels of coagulation factors or different activation rates with significant modifications in the thrombus formation rates [89,90,91,92]. Other risk factors, deriving from rare conditions, can give rise to occlusions or severe reduction in myocardial perfusion. These include vasculitis that can cause coronary occlusions, ventricular hypertrophy, production of emboli following trauma to coronary arteries, coronary artery abnormalities (including aneurysms, aortic dissection), severe anemia, or pulmonary infection. Furthermore, not only a reduced oxygen supply to the myocardium can cause ischemia, but also an increased demand, due to hypothyroidism, fever, and heavy exertion [85,87]. Moreover, restoration of blood flow after a period of ischemia can cause potentially very harmful effects such as necrosis of damaged cells, marked cell swelling and restoration of non-uniform flow in all portions of the tissue. The latter, called the reflux phenomenon, is the result of a vicious cycle of vascular, endothelial, and mitochondrial dysfunctions, with reduced local perfusion, major dysfunctional changes, edema, and much more. All these functional and structural changes are referred to as ischemia/reperfusion (I/R) injury [93].

Although in mammals CMs contain large stores of energy phosphate, in in vivo models, following ischemia, the onset of systolic dysfunction is much more rapid than expected. This is because the breakdown of the creatinine phosphate molecules leads to the production of inorganic phosphate which inhibits the proteins with contractile function. Furthermore, contractility is inhibited by calcium depletion following intracellular acidosis. The blockage of CMs’ aerobic metabolism induces a rapid reduction of ATP and a concomitant increase of anaerobic metabolites, such as lactate. If this block is maintained for less than 10–20 min, cardiac functions can be totally reversible, while if its duration is longer, the damage becomes irreversible and CMs are no longer able to maintain their structural integrity [87].

### 2.1. Inflammation Phase

During the very early stages following ischemic injury, MCs and soluble complement proteins become important initiators of inflammation [94], leading (in the absence of a rapid reconstitution of coronary blood flow) to loss of CMs, which undergo cell death by apoptosis, necrosis, and necroptosis. The former includes the extrinsic or death receptor pathway and the intrinsic or mitochondrial pathway, although it has been shown that the two are linked and mutually influence each other [95]. The extrinsic and intrinsic apoptotic pathways join into a common trail starting with caspase-3 cleavage up to DNA fragmentation, degradation of cytoskeletal and nuclear proteins, proteins cross-linking, expression of ligands for phagocytic cell and apoptotic bodies formation. The latter are then phagocytosed by macrophages and/or the neighboring cells before they fragment [96].

Among the different types of cell death, necrosis is an uncontrolled form of cell death due to sudden damage, such as a hypoxic event. It is characterized by the swelling of inner cell structures, rupture of plasma membrane up to the complete lysis of the cell and release of intracellular contents leading to tissue damage [97]. Finally, necroptosis is activated by death receptors but determines the production of reactive oxygen species (ROS) and the depletion of ATP, as in necrosis. However, it remains to be clarified how these three cell death mechanisms interact with each other during the ischemic event [98]. On the contrary, what is well known is that necrotic cells release endogenous damage-associated molecular patterns (DAMPs), that can be recognized by pathogen recognition receptors (PRR) and Toll-like receptors (TLRs), a family of transmembrane receptors that activate downstream pro-inflammatory cascade and recruit neutrophils, macrophages, monocytes, MCs and dendritic cells (DCs) [4,87]. After the blood flow has been restored, the release by the cardiac resident MCs of preformed pro-inflammatory mediators, such as TNF-α, stimulates the CMs apoptosis, and the release of histamine and various proteases, which amplify the inflammatory signal involving ECs, resident macrophages, and subsequently infiltrating monocytes [4,94]. Finally, the activated complement system contributes to inflammatory signaling by accentuating CMs necrosis, inducing proinflammatory responses and mediating leukocyte influx in the injured myocardium [87]. 

Neutrophils accumulate in large numbers in damaged tissue in the first hours after ischemia, localizing in post-capillary venules. In the first phase, they are activated before infiltrating damaged tissue. Following the release of formylated peptides and mitochondrial DNA, detected, respectively, by formyl peptide receptor 1 (FPR1) and TLR9, there is not only the activation of neutrophils but their chemotaxis towards the damaged area, with consequent infiltration [99]. The second phase of neutrophil recruitment involves cardiac ECs, activated by cytokines such as TNF-α and histamine, resulting in increased expression of selectins. The interaction between selectins and their receptors allows neutrophils to roll along the venule wall, where they more easily meet activating factors, such as IL-8 and C5a. These interactions lead to neutrophil integrin activation, which binds to members of the immunoglobulin superfamily expressed in stimulated ECs and allow the firm adhesion of the leukocytes. Trans endothelial migration follows and leads to neutrophil infiltration in the inflamed tissues [100,101], where they begin to engulf the dead or dying CMs, thus reducing the presence of pro-inflammatory DAMPs. At the same time, neutrophils cause the release of oxygen free radicals, inflammatory mediators, and proteolytic enzymes, such as metalloproteinases (MMPs), resulting in degradation of the ECM and cardiac rupture essentially in the absence or severe reduction of expression of *F13A1* gene as reported in a mice animal model [102]. The causal relationship between MMP-9 levels and heart rupture in infarcted myocardium has been established by Heymans in *KO* mice for the *MMP9* gene which protects against cardiac rupture [103]. This was further corroborated by the observation that plasma FXIIIA levels significantly drop in the first hours after MI [104], and appropriate circulating levels of FXIIIA molecules or *F13A1* gene variants can contrast the unrestrained MMPs action in MI patients [105,106,107], or in fibroblast cell culture adjuvating wound healing in several chronic skin lesions [108,109,110].

MCs and mast cell-committed progenitors (MCPs) recruited in the ischemic cardiac region following inflammatory triggers seem to come mostly from white adipose tissue (WAT), where stem cells with multilineage properties are contained. Once infiltrated in cardiac tissue, WAT-derived MCPs proliferate and differentiate into mature cells in an SCF-dependent manner, they can therefore directly regulate CMs contractility as well as produce cytokines for monocyte recruitment [111].

Among the early mediators of tissue injury, IL-6 produced by CMs and by recruited myeloid cells plays a pivotal role by upregulating intercellular adhesion molecule 1 (ICAM-1) on CMs leading to neutrophil binding and stimulation of their cytotoxic activity [94]. IL-6 and TNF-α contribute to the expression and production of the chemokine CCL2 [112] by mainly ECs and infiltrating leukocytes, with the function of recruiting, activating, and differentiating monocytes and macrophages expressing the CCR2 [113]. Suppression or reduction of this chemokine appears to attenuate adverse remodeling following MI [114]. Some adhesion molecules, such as vascular cell adhesion molecule 1 (VCAM-1), contribute to monocyte recruitment by binding the integrin very late antigen-4 (VLA-4) on their cell surface. Pro-inflammatory Ly-6C^high^ monocytes, also defined as M1-type, dominate on days 1 to 4 and promote phagocytosis of apoptotic and necrotic myocytes and neutrophils and other debris, release pro-inflammatory mediators such as TNF-α, IL-1b, IL-6 and macrophage inflammatory protein 1 alpha (MIP-1α), and molecules able to digest preexisting collagen network, like myeloperoxidase, MMPs, cathepsins, and plasminogen activator urokinase [115]. In the early stages following ischemia, monocyte macrophages also begin to produce macrophage-colony stimulating factor (M-CSF), which stimulates phenotypic changes of monocytes proliferation and differentiation into mature macrophages, in an autocrine mechanism, responsible for macrophages growth and activation [116]. It is therefore possible to state that cardiac tissue-resident macrophages drive monocyte recruitment, fate specification, inflammation, and adverse left ventricular (LV) remodeling [10]. Interestingly, FXIIIA is contained in macrophages and platelets, linking in turn coagulation, thrombosis, inflammation, and healing processes [92,117]. 

Similarly, cardiac resident fibroblasts presenting TLRs can easily respond to DAMPs produced by CMs necrosis, generating granulocyte-macrophage colony-stimulating factor (GM-CSF) involved in the production of neutrophils and monocytes, which follow chemotactic signals, such as CCL2 (for CCR2+ monocytes) and CXCL2 (for neutrophils), up to the infarcted myocardium [118]. Fibroblasts may modify their functions following myocardial insults with changes in the secretion of cytokines and growth factors acting in a paracrine and/or autocrine manner altering protein expression, cell proliferation and cell migration. In all post-ischemia stages, fibroblasts activated by TGF-β and other factors, control changes in the ECM. The phenotypic conversion into myofibroblasts, obtained following activation, leads to increased fibrin production, and persistent myofibroblast proliferation leads to cardiac remodeling [2]. 

Inflammation results, therefore, as a key pathophysiologic process of MI, in common with other CV diseases, and is potentially involved in disease progression, severity and clinical outcome [7,8,119,120,121]. At this point, it is natural to wonder what mechanisms initiate the resolution of the infarct inflammation since temporal and spatial containment of inflammation is a prerequisite for arrayed wound healing and prevention of adverse ventricular remodeling. In recent years, much attention has been paid to the role of regulatory T cells (Treg) as effectors of the switch between the inflammation phase and the resolution phase of MI [122,123,124,125,126].

### 2.2. Epigenetic Regulation

The inflammatory response to MI is in part regulated by at least one active histone modification, such as H3K9ac, H3K27ac and H3K4me3. These three marks, mainly present at the promoter level, regulate the expression of chemokines essential in the early stages of MI, such as CCL2, or of proteins involved in the development and differentiation of CMs [127]. CMs survival is also promoted by histone acetylation stimulated by acetyl-CoA synthesis from sodium octanoate (8C) metabolism [128]. HDACs are also involved in the first post-MI phase, such as Sirtuin 2 (SIRT2), a class III HDAC, which is able of deacetylating both histones and numerous non-histone proteins, such as DNA transcription and repair factors [129,130]. It has been reported that SIRT1 is involved in endothelium-dependent vasodilation, via the deacetylation of lysines 496 and 506 of the calmodulin-binding domain of the endothelial nitric oxide synthase (eNOS), leading to enzyme-increased activation and increased endothelial nitric oxide [131]. The latter, unlike its vasodilator effect, has negative results, such as S-nitrosylation and therefore inhibition of SIRT1 [132], which instead seems to possess cardioprotective effects. Sirtuin 1 indeed increases the expression of molecules such as MnSOD, TrX and Bcl-xL while reducing that of pro-apoptotic factors such as Bax, which is positively regulated by p53, which in turn is deacetylated and then inhibited by SIRT1. Sirtuin 1 likewise deacetylates FoxO by stimulating the expression of cell-protective genes [133].

**Figure 5:**
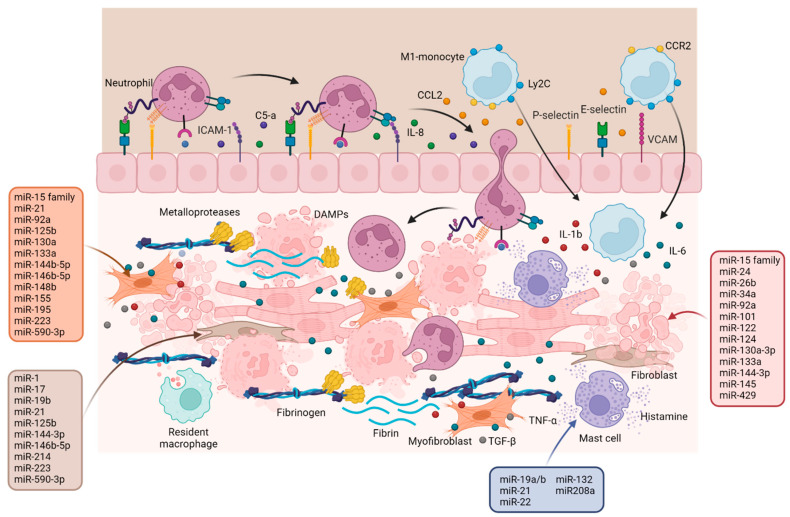
**The inflammation phase.** Schematic representation of the key cell subsets involved in the post-MI inflammatory phase, including neutrophils and M1-macrophages, as well as pro-inflammatory cytokines and chemokines. miRNAs regulate various aspects of this phase: cardiomyocyte apoptosis, inflammation, and fibrosis, both by activating CFs and by stimulating the fibroblast-to-myofibroblasts switch. The miRNAs involved in the regulation of the different processes are listed in the boxes. Created with BioRender.com.

Nonetheless, the most studied epigenetic factors regulating post-MI mechanisms are miRNAs, which mainly act in a synergistic or antagonistic way on the key processes occurring after ischemic damage. Figure 5 and Table S1 show the main miRNAs that are presumably involved in the inflammatory phase stratified according to the mechanism they regulate: (i) cardiomyocyte apoptosis; (ii) inflammation; (iii) fibrosis.

In the first case, the miRNAs are pro- and anti-apoptotic, based on their ability to inhibit genes activating apoptosis, such as miR-133a [134], or genes involved in anti-apoptotic processes, such as miR-124 [135]. Many of these miRNAs show overlapping effects, although they are included in different signaling pathways. For example, miR-34a which blocks the expression of aldehyde dehydrogenase 2 (ALDH2), involved in alcohol metabolism [136], and miR-429 which downregulates the transmembrane receptor Notch1 [137]. In addition, a miRNA can target different proteins with the same result, as for the anti-apoptotic miR-101 [138,139].

In the case of miRNAs that can regulate inflammation and oxidative stress, most of them appear to be involved in the suppression of histone deacetylase SIRT1 (miR-29a [140], miR-132 [141], miR-155 [142]), which is downregulated following I/R damage, with consequent reduction of its cardioprotective effect.

Finally, the activation of CFs and their conversion into myofibroblasts is an important aspect of the inflammatory phase, on which the progression of myocardial damage or its resolution will depend. Most miRNAs seem to have pro-fibrotic effects, which allow for the proliferation, migration, and differentiation of CFs, as well as the production of collagen and ECM, except four with anti-fibrotic function: miR-130a [143], miR-133a [144], miR-148b [145] and miR-590-3p [146]. However, the main pathway regulated by pro- and anti-fibrotic miRNAs is TGF-β signaling and its receptors such as TGFbR and Smads, apart from miR-21 [22], miR-92a [147] and miR-195 [148] which all inhibit Smad7. In turn, TGF-β activates other pathways such as PI3K/AK and JAK- STAT signaling [149], which in turn are targeted by different miRNAs involved in heart healing.

## 3. Resolution Phase 

The suppressor Treg cell activation, directly promoted by the heart and its draining lymphonodes, alleviates local inflammation, protects CMs from apoptosis, enhances M2-like monocyte differentiation, which promotes wound healing, angiogenesis, fibrotic, and scavenger processes, and modules myofibroblasts activation [4,122,124,126]. Treg cells produce TGF-β, IL-10, and IL-13, which stimulate anti-inflammatory macrophage activation, repress anti-inflammatory signals, and stimulate neo-angiogenesis. The activated Treg cells also induce an M2-polarizing milieu locally within the heart, to which IL-10 and TGF-β produced by M2-like macrophages themselves contribute, while TGF-β and IL-13 work together to stimulate collagen deposition by myofibroblasts. Collagen production is also influenced by osteopontin, whose release from M2-type monocyte is stimulated by TGF-β, IL-10 and IL-13 [122]. In addition to the production of collagen by myofibroblasts and fibrin deposition, the presence of FXIII, which promotes cross-linking between fibrin and ECM components, is extremely important for the integrity and elasticity properties of the scar tissue. FXIII is also essential for other important tasks, such as the recruitment of adult stem cells and neo-angiogenesis by inhibition of the potent anti-angiogenic factor thrombospondin-1 (TSP-1) [150,151], so that dynamic of FXIIIA during the first hours after MI can be considered a prognostic indicator of the infarct evolution [104,122].

Treg lymphocytes have numerous influences on the cells present in the injured myocardium, which allow for the resolution of inflammation and the repair of ischemic damage. In fact, they inhibit cytokine production by fibroblasts; promote angiogenesis by acting on ECs; slow down cytokine production and apoptosis as well as promote the proliferation of CMs; inhibit the production of cytokines by Th1/Th17/CD8+ T lymphocytes and of cytokines and antibodies by B cells, as well as T cells migration and B cells proliferation [125].

In addition to the presence and action of Treg lymphocytes, an essential step for the transition to the resolution phase is the removal of debris produced by the degradation of the ECM and apoptotic cells, including CMs and neutrophils that are programmed to undergo apoptosis [87]. They are phagocytosed by macrophages, leading to the release of anti-inflammatory cytokines as well as anti-inflammatory and pro-resolving lipid mediators as lipoxin A4, resolving E1 and protectin D1. The latter contribute to abrogate neutrophil influx in the injured myocardium through the production of lactoferrin, an anti-inflammatory glycoprotein that specifically inhibits chemotaxis of neutrophils but not mononuclear phagocytes [152]. Finally, phagocytic clearance of apoptotic neutrophils reprogrammes monocyte-derived macrophages from a pro-inflammatory (M1-type) to an anti-inflammatory (M2-type) phenotype [153]. 

M2-type macrophages are widely present in infarcted myocardium and thanks to the secretion of anti-proliferative cytokines and mediators that terminate pro-inflammatory IL-1 signaling, such as the decoy type II IL-1 receptor and IL-1 receptor antagonist (IRA), they can be considered among the undisputed protagonists of the resolution phase [87]. Furthermore, local upregulation of M-CSF creates a microenvironment suitable for macrophage growth, differentiation, and survival. M-CSF can also promote cardiac repair by modulating the endothelial cell phenotype, stimulating the production of the monocyte chemoattractant protein-1 (MCP-1), which, in addition to inducing monocyte chemotaxis, promotes angiogenesis. This may be due either to the induction of production of growth factors such as vascular-endothelial growth factor (VEGF) or by directly acting on proliferation of the ECs [116,154].

Among the anti-inflammatory cytokines produced by macrophages and Treg lymphocytes, TGF-β plays an important role since it attenuates trans-endothelial leukocyte migration, suppresses the production of pro-inflammatory chemokines and cytokines, enhances M-CSF-induced proliferation, inhibits nitrite release, reduces cytotoxic activity, and regulates the phenotype and functions of the T lymphocyte subpopulations [155]. TGF-β also stimulates massive proliferation of myofibroblasts, which become the predominant cell type in healing infarct, as well as the preservation of the ECM, through the production of ECM proteins, such as collagen I, collagen III and fibronectin, the suppression of collagenase expression and the induction of metalloproteinase inhibitor (TIMP-1) secretion [155,156].

During the resolution phase, the ECM is enriched by the presence of matricellular proteins, a family of structurally unrelated extracellular macromolecules that interact with surface receptors, growth factors, proteases, but also with structural matrix proteins, without having a direct structural role. They act as bridges between cells and matrix, integrating signals that modulate cellular behaviour. Matricellular proteins, including TSP-1 and TSP-2, SPARC (secreted protein acidic and rich in cysteine), tenascin-C, osteopontin, and periostin, are usually poorly expressed in the healthy heart, while they are strongly upregulated following cardiac injury, where they perform the function of transducers of key molecular signals in cardiac repair and modulators of cell migration, proliferation, and adhesion. They also modulate cytokine and growth factor signaling, regulate matrix assembly and metabolism, and modulate the fibrogenic potential of inflammatory cells and fibroblasts [157]. The latter contribute to the matrix metabolism, producing cytokines, growth factors, matricellular proteins and MMPs [155].

ECs make up the largest percentage of non-cardiomyocytic cells in the heart [158] and play important roles in the various stages following ischemic damage, restoring oxygen and nutrient supply [159]. Neo-angiogenesis is an integral and essential part of wound healing and, already a few hours after ischemic damage, growth factors such as basic fibroblast growth factor (bFGF) and VEGF stimulate the angiogenic activation of ECs and the formation of new vessels. During the proliferative phase, enlarged vessels are formed, with thin walls poor in pericytes, called “mother vessels”, with transluminal projections capable of dividing the vascular structure into multiple lumens. The absence of pericytes makes these vessels more plastic and therefore capable of adapting to continuous tissue changes. Over time, mother vessels evolve into smaller daughter vessels, such as medium-sized muscular arteries and veins [160]. Many other factors, such as TGF-β, MCP-1, IL-8 and IL-10 are involved in the regulation of angiogenesis and may participate in the formation of new vessels after cardiac infarction. The composition of the ECM is also critical for a dynamic modulation of vascular growth, especially the presence of collagen [87,156]. The key role of cytokines/chemokines/growth factors on endothelial homeostasis and tissue repair suggests that they may be targeted for therapeutic purposes as demonstrated in other complex vascular diseases [161,162].

### Epigenetics Regulation

Since myofibroblasts are primarily responsible for normal wound healing and tissue repair after MI, it is very important that they are highly regulated to prevent their excessive activation, which could lead to pathological fibrosis, or reduced ECM deposition which can cause poor repair or detrimental myocardial wall rupture. On the other hand, excessive activity or long-lasting of fibrotic constituents, may result in a permanent damage of the reparative tissue with loss in efficiency of the elastic components as demonstrated in other tissue modelling [163]. As a result, molecule involved in the crosslinking of ECM components, as *F13A1* gene product, is predicted to be targeted by at least 133 different miRNAs as reported in the miRDB database (http://www.mirdb.org, accessed on 26 July 2023) suggesting a complex and articulate epigenetic regulation and epi-druggable target. 

Moreover, hyperacetylation of histone H4 at the collagen type 1-alpha-2 locus by p300 facilitates TGF-β-induced collagen production by myofibroblasts. Conversely, HDAC4-mediated deacetylation is associated with TGF-β-mediated suppression of collagen production and increased expression of repressors of the TGF-β1 signaling pathway [164]. HDACs are generally upregulated during fibroblast activation, a hypothesis supported by the beneficial effects on remodelling of deacetylase inhibitors [165]. Class I and II HDACs seem to be largely involved in the induction of cardiac hypertrophy, through the repression of antihypertrophic genes, such as Krupple-like factor 2 (Klf2) [166]. HDAC2 seems to induce cardiac hypertrophy by increasing the Akt signaling pathway, which is involved both in cardiac hypertrophy and in the maintenance or improvement of cardiac functions [167,168].

Class III HDACs have opposite protective effects towards CMs as SIRT1, whose expression is upregulated in stressful situations such as inflammation, inhibiting both hypertrophy and apoptosis following oxidative stress [169]. Indeed, it has been demonstrated that the production of ROS is an essential mechanism for the development of cardiac hypertrophy and that SIRT3 is able to control its accumulation, consequently blocking the activation of pathways, such as MAPK/ERK and PI3K/AkT, involved in cardiac hypertrophy [170]. SIRT6 in turn, also acts as a negative regulator of cardiac hypertrophy reducing the differentiation of CFs into myofibroblasts, through suppression of the transcriptional activity of NF-kB, a key regulator of the inflammatory reaction involved in the development of cardiac remodeling [171]. DNA methylation is also involved in the regulation of MI effects, particularly in cardiac fibrosis. In fact, it has been seen that in hypoxic conditions CFs express hypoxia-inducible factor 1-alpha (HIF-1α) which in turn determines the upregulation of the *DNMT1* and *DNMT3a/3b* genes with consequent alteration of DNA methylation and activation of pro-fibrotic genes [165].

**Figure 6:**
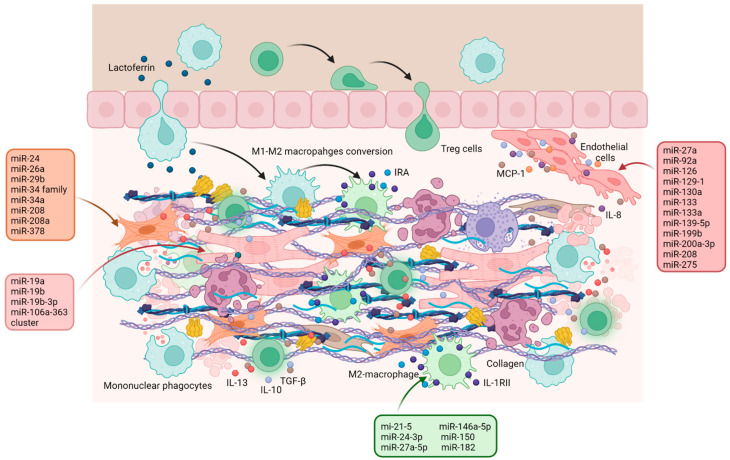
**The resolution phase.** Schematic representation of the main events of the resolution phase regarding Treg recruitment, the anti-inflammatory polarization of macrophages, the release of anti-inflammatory cytokines, the deposition of ECM and the initiation of neo-angiogenesis. The miRNAs involved in the regulation of the different processes are listed in the boxes. Created with BioRender.com.

HIF-1α is also involved in angiogenesis and is negatively modulated by miR-126 [172] (Figure 6 and Table S2), although miRNAs that appear to regulate new blood vessel formation following MI preferentially target VEGF and its receptor VEGFR, such as miR-129-1, miR-133, miR-139-5p, miR-199b and miR-200a-3p [173,174,175,176]. In addition, some miRNAs act on the proliferation and migration of ECs, regulating the PI3K/Akt signaling pathway (miR-130a and miR-208 [177,178]).

Even in the resolution phase, regulation of cardiac fibrosis is important to prevent adverse consequences such as LV remodeling and heart failure [7,8]. As conceivable, miRNAs likely to act on fibrosis at this stage mainly regulate the TGF-β signaling pathway or directly the expression of collagen genes in myofibroblasts or activated CFs. For example, miR-26a appears to directly target collagen type 1 (COL1) and connective tissue growth factor (CTGF) [179], while miR29b [180] and miR-208 [181] inhibit COL1 expression, by downregulating adapter protein SH2B3 and transcription factor GATA4, respectively. However, miR-29b is also able to directly target the expression of fibrosis-related genes, such as *COL1A1* and *COL3A1* [182].

A very important aspect of the resolution phase is the polarization of macrophages from the pro-inflammatory to the anti-inflammatory phenotype. This step is essential to limit the progression of inflammation and proceed towards its resolution and although its regulation largely depends on the production of cytokines such as TGF-beta, IL-10, IL-13 and M-CSF [116,122,154], in recent years several studies on non-coding RNAs have been performed.

Although these investigations are still in their infancy, most of the miRNAs analyzed appear to come from exosomes released from mesenchymal cells and CMs and seem to act primarily by regulating the PI3k/Akt signaling pathway (such as miR-21-5p, miR-150 and miR-182), or the NF-kB pathway (namely miR-24-3p) [183,184,185,186]. Unfortunately, there are few miRNAs that can stimulate the re-entry of CMs into the cell cycle, allowing their proliferation and differentiation, with consequent repopulation of the damaged region and these include miR-19a, miR-19b and miR-106a-363 cluster [34,187].

Among the various miRNAs studied, one seems to regulate several aspects of the resolution phase, even if it targets only one pathway: miR-375 regulates the PDK-1/Akt signaling, promoting neovascularization, reducing cardiomyocyte apoptosis and inducing the switch from M1- to M2-macrophage, all actions that limit the expansion of the infarct area and the dysfunction of the left ventricle [188].

## 4. Maturation Phase

The formation of a complex network of ECM proteins and the deposition/cross-linking of collagen molecules [189] determine the end of the proliferative phase and the beginning of the maturation phase, with consequent production of a stable scar [190]. Different data have been reported concerning the fate of myofibroblasts during this phase. Initially, it was thought that the latter undergo some mechanisms which determined their loss at the end of the physiological tissue repair: cell release from stress, the increase of cross-linking between matrix molecules and an increased formation of specific cell-cell contacts, up to their death by apoptosis [191]. Recently, it has been shown that both activated fibroblasts and myofibroblasts persist at a 3.5-fold higher concentration in the infarcted region than in a healthy heart, over a long period of time without cell turnover. The scar that forms in this last phase must persist in the long term due to the impossibility of the adult heart to regenerate and hosts highly differentiated fibroblasts adapted to a deeply fibrotic environment, matrifibrocytes, which have the task of maintaining the integrity of the mature scar over time [192].

Another important change that occurs during maturation is the coating of new blood vessels with mural cells, such as pericytes and vascular smooth muscle cells (VSMC), which stabilize the microvasculature of the scar and attenuate inflammation, preventing extravasation of blood cells, while uncoated vessels regress [190,193]. The regulation of the two major events associated with scar formation, collagen deposition and vasculature maturation, is largely dependent on platelet-derived growth factor (PDGF) signaling. Both PDGFR-α and PDGFR-β receptors mediate fibrogenic effects, but only the latter is involved in the regulation of microvessel coating [194]. PDGFR-β is expressed by pericytes and vascular smooth muscle cells (VSMCs), while ECs secrete PDGF, which mediates mural cell proliferation and migration [195]. Alteration of this signaling pathway can lead to continued inflammation and haemorrhage and adverse cardiac remodeling.

### Epigenetics Regulation

As already highlighted in Figure 3, the Notch pathway can stimulate the proliferation of CMs in the pre-natal period, while it is blocked shortly after birth and cannot be reactivated following MI due to epigenetic alterations at the level of its promoter. This region is in fact modified with the H3K27Me3 mark, associated with chromatin condensation and gene silencing [196]. Also, histone acetylation, as already stated, and CpG island methylation are involved in the silencing of genes that allow the reactivation and proliferation of CMs and could therefore be considered potential therapeutic target to improve the outcomes of MI [197].

**Figure 7:**
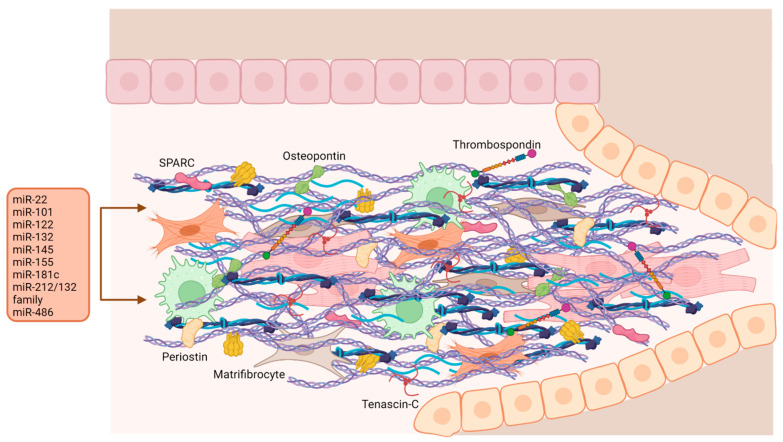
**The maturation phase.** Schematic representation of the maturation phase in which the ECM is stabilized with the release of collagen and matrix proteins, the neo vessels are coated with mural cells, to strengthen them, and part of the fibroblasts are transformed into matrifibroblasts involved in the scar stability. Adverse outcomes such as cardiac remodeling are regulated at this stage by miRNAs. Created with BioRender.com.

The miRNAs presumably involved in the maturation phase mainly regulate cardiomyocyte hypertrophy, vascular smooth muscle cell differentiation, myofibroblast senescence and, more generally, cardiac dysfunction, remodelling and damage (Figure 7 and Table S3).

Is it possible that the mechanisms and processes that took place in the two previous phases have already decided the fate of the infarcted myocardium? In 2010, Nahrendorf had proposed that the very first days immediately following the heart ischemic injury determine the fate of the patient [115]. This view has been confirmed by contemporary CV therapy showing that it is possible to reverse the LV remodelling with a consequent improvement in myocardial function, up to a total recovery in rare cases, but only if timely intervention is performed after MI establishment [198].

## 5. Sex-Related Differences in Risk Factors and Long-Term Outcomes for MI

Males and females have marked differences in the risk factors, symptoms, and outcomes of MI. The most well-known cardiovascular risk factors appear to be differently associated with a greater risk of developing MI in the two sexes [86].

Smoking, for example, is associated with higher risk in women and increases with the number of cigarettes consumed. Type 1 and type 2 diabetes, as well as atrial fibrillation, also lead to an increased risk of ischemic damage in females, although the incidence of MI is less than half that in males [199,200]. Unfortunately, females have additional sex- and gender-specific risks, including early menopause and pregnancy-related disorders [201,202,203]. In postmenopausal females, specific independent predictor for increased risk of MI have been recognized in MMP mediated collagen degradation products [204]. Females with a history of gestational hypertension and preeclampsia have a 60% greater risk of developing CV diseases, and this persists into their 70s [205]. Increased blood glucose content during pregnancy, even without the development of gestational diabetes, also has a strong impact on the risk of MI [206]. Moreover, regarding the symptoms of MI there are significant differences between the two sexes reporting a greater number of symptomatic phenotypes and some of these not specific for MI [207]. Although females report chest pain more often and earlier after ischemia than males, it is usually a non-specific pain that is unlikely to be related to ischemic damage [208]. In addition to the difficulty in recognizing symptoms (by both females themselves and by health care specialists), other social aspects may contribute to a marked delay in presentation and revascularization in females with acute MI. Among these, the deep-rooted idea that female sex is rarely affected by ischemia and the women social role which relegates women to providing assistance rather than receiving it [201].

Post-MI outcomes and complications are also influenced by sex differences. First, females experience longer hospitalizations, more bleeding complications, and higher in-hospital mortality following coronary revascularization than the sex counterpart. Furthermore, females have an increased risk of early death, complications such as heart failure and cardiogenic shock, and recurrent MIs. The latter could be related to several factors. Compared to men, women are more likely to have non-obstructive atherosclerotic coronary artery disease or spontaneous coronary artery dissection, a different coronary physiology with increased plaque vulnerability, a smaller coronary luminal area regardless of body size, with larger susceptibility to thrombotic occlusion, and worse outcomes after revascularization [209].

Is it possible that these anatomical differences alone underlie the differences in risk factors, symptoms, and outcomes between females and males? Might there be an extensive different epigenetic regulation of the various stages of MI involved in the two sexes? Epigenetic modifications of DNA and histones, as well as non-coding RNAs, have been studied mainly in cells and animal models, so is it possible that there are substantial differences in sex-related epigenetic regulations? And how could this affect the development of targeted efficient therapeutic strategies?

Overall, the number and the complexity of the clinical unmet needs, ask for appropriate and dedicated answers, and this is worthily approached by the international scientific community in several complex diseases by considering both genetics and epigenetics aspects [86,210,211,212].

## 6. Conclusions

In the present review, the main post-MI processes and the epigenetic factors that govern them have been reported, to present a global idea of the heart healing process and its regulation. Epigenetic control mechanisms can have synergistic, additive, or antagonistic effects, and the regulation of a single physiological or pathological phenomenon is therefore very intricate to decipher. Even in the case of the epigenetic regulation of pre- and post-MI cardiac processes, the combined action of many different factors makes it extremely difficult to intervene in order to have a significant effect on the course of symptoms. Just think of the number of all the miRNAs involved in the proliferation of CMs or in the regulation of cardiac fibrosis.

Many research groups are currently concentrating on identifying molecules that can either block or mimic the effects of miRNAs, which are being considered as potential therapeutic markers. However, there have been no studies that have progressed beyond animal testing. Moreover, in some cases, the effects of these non-coding RNAs have been known for a decade now, though dedicated clinical trials on humans have not yet been started. Interestingly, the three anti-miRs that were in the pipeline by miRagen Therapeutics in 2017 (MGN-4220 against miR-29 for the treatment of cardiac fibrosis, MGN-1374 able to inhibit miR-15 and miR-195 for the treatment of post-MI cardiac remodeling, and MGN-9103 which targets miR-208, for the treatment of chronic heart failure) [213], are interesting candidates to test. Certainly, one of the main obstacles is the difficulty in the delivery of RNAs, easily degraded by RNases present in body fluids, but on which significant goals are being achieved [214]. Another difficult problem to deal with is the complex regulation of the various myocardium processes. It is not only important to understand the function of epigenetic factors and verify their up or down regulation following MI, but also to verify how they vary in subjects who experienced MI with different outcomes. Several important questions remain to understand what exactly allows one individual to completely recover after MI compared to the majority of patients. What changes between subjects who have undergone fibrosis or LV remodeling? The comparison of the miRNA expression profiles, or other epigenetic factors will allow us to identify key element in heart healing. Precision medicine fits right into this context, being able to verify in the period immediately following the ischemic damage how miRNAs vary, allowing for effective intervention.

## Figures and Tables

**Table 1:** Cardiomyocytes regulation: mechanistic insights on pro-proliferative miRNAs.

miRNA	Model	Targets	Pathway	Effects	Mechanism(s)	Ref.
miR-10b	Hu	Lats1	Hippo	↓apoptosis	miR-10b blocks the expression of Lats1 and thereby inhibits the Hippo pathway	[45]
miR-17-92	M; R	PTEN	Akt	↑proliferation	miR-17-92 represses PTEN-inducing cardiomyocyte proliferation mediated by the AKt signaling pathway	[33]
miR19a/19b	M	PTEN	Akt	↑proliferation	miR-19a/19b blocks the expression of PTEN inducing cardiomyocyte proliferation mediated by the AKt signaling	[34]
miR-21-5p	Hu	PDCD4/ PTEN	Akt	↑proliferation	miR-21-5p directly represses PCDN4 e PTEN-inducing cardiomyocyte proliferation mediated by the AKt signaling	[46]
miR-24	R	p27	Cell cycle	↑proliferation	miR-24 promotes cells in G0/G1 phase into S phase by repressing p27 expression	[47]
miR-25	Hu; Zb	FBXW7	Cell cycle	↑proliferation	miR-25 inhibits FBXW7, a cell-cycle regulatory factor that mediates the proteolysis of positive cell-cycle regulators	[48]
miR-101a	Hu	TGFBR1	MAPK	↑proliferation	miR-101a blocks the expression of TGFBR1 and thereby activates the MAPK signaling pathway	[49]
miR-145-5p	R	GIGYF1	/	↓apoptosis	miR-145-5p inhibits GIGYF1, a regulator of mRNA turnover, blocking translation and activating transcript decay	[50]
miR-152	M	p27	Hippo	/	The increase of miR-152 by the activation of YAP1 represses the expression of p27	[51]
miR-199a-3p	R	CD151	MAPK	↑proliferation	miR-199a-3p inhibits the tetraspanin CD151, involved in several cellular process	[52]
miR-199a-3p	R	TAOK1	Hippo	↓apoptosis	miR-199a-3p represses Serine/threonine-protein kinase TAOK1 involved in the Hippo pathway	[53]
miR-199a-3p; -302d; -373; -590-3p; -1825	R	STK38L	Hippo	↓apoptosis ↑proliferation	They downregulate STK38L, a direct targeting on the 3’UTR of the related mRNA has not been identified	[53]
miR-200a-3p	Hu; M	PDCD4	Akt	↑proliferation	miR-200a-3p represses PCDN4 activating AKt signaling	[54]
miR-204	R	Jarid2	/	↑proliferation	miR-204 downregulates Jarid2, a non-catalytic member of the polycomb repressive complex 2	[42]
miR-210	M	APC/β-catenin	WNT	↑proliferation	miR-210 represses the cell cycle inhibitor APC, involved in the WNT pathway	[55]
miR-221-3p	R	PTEN	Akt	↑proliferation	It enhances Akt kinase activity by inhibiting PTEN	[35]
miR-294	M	Wee1	Cell cycle	/	miR-294 inhibits Wee1, increasing the activity of the cyclin B1/CDK1	[56]
miR-301a	M; R	PTEN	Akt	↑proliferation	PTEN is a direct target of miR-301a in regulating cardiomyocyte proliferation	[57]
miR-302d; -373; -590-3p	Hu	LATS2	Hippo	↓apoptosis ↑proliferation	miR-302d, miR-373 and miR590-3p target LATS2, inhibiting the Hippo pathway	[58] [53]
miR-324	Hu	SOCS3	NFkB	↑proliferation ↓apoptosis	miR-32a represses SOCS3, activating the NFkB pathway	[38]
miR-362-3p	R	TP53INP2	SDF1/ CXCR4	↑proliferation ↓apoptosis	miR-362-3p downregulates TP53INP2, activating at the same time the SDF-1/CXCR4 pathway	[59]
miR-374	M	DTNA	Notch	↑viability ↓apoptosis	miR-374 inhibits DTNA and the Notch1 axis	[60]
miR-410; -495	M; R	Cited2	Cell cycle	↑proliferation	miR-410 and miR-495 downregulate the transcriptional coactivator Cited2	[61]
miR-410-3p	Hu	TRAF5	NFkB	↓apoptosis	miR-410-3p protects CMs from apoptosis by repressing TRAF5 expression	[62]
miR-496	R	HOOK3	Akt	↓apoptosis ↑proliferation	miR-496 binds to Hook3 to inhibit the activation of PI3K/Akt/mTOR signalling pathway	[63]
miR-499	M; R	SOX6	Cell cycle	↑proliferation ↓apoptosis	miR-499 promotes cell proliferation and inhibits apoptosis in the late stage of cardiac differentiation targeting SOX6 and leading to activation of cyclin D1	[29]
miR-708	M; R	MAPK14	MAPK	↑proliferation	miR-708 inhibits the expression of MAPK14, which arrests the cell cycle in CMs	[64]
miR-877-3p	M	GADD45g	Cell cycle	↑proliferation	LED-Red irradiation increases miR-877-3p expression promoting proliferation of neonatal CMs via GADD45g	[65]
miR-1180	R	NKIRAS2	NFkB	↑proliferation ↑viability ↓apoptosis	miR-1180 represses the NF-κB inhibitor interacting with Ras-like 2 (NKIRA2), activating the NFkB pathway	[39]

Abbreviations: Hu: human; M: murine; R: rat; Zb: zebrafish.; ↑: upregulation/increase; ↓: downregulation/decrease.

**Table 2:** Cardiomyocytes regulation: mechanistic insights on anti-proliferative/pro-apoptotic miRNAs.

miRNA	Model	Targets	Pathway	Effects	Mechanism(s)	Ref.
Let-71-5p	M	E2F2–CCND2	Cell cycle	↓proliferation	Let-7i-5p downregulates the expression of E2F2 and CCND2 and further represses CM proliferation	[66]
miR-1	M	CCND1	Cell cycle	↓proliferation	It directly suppresses the cell-cycle regulator CCND1	[67]
miR-1	R	NOTCH3	Notch	↑apoptosis ↓proliferation ↓autophagy	miR-1 suppresses NOTCH3 thereby negatively regulating the Notch pathway	[68]
miR-9	R	YAP1	Hippo	↑apoptosis	miR-9 promotes hypoxia-induced cardiomyocyte apoptosis by targeting Yap1	[69]
miR-26a	R, Zb	Ezh2	/	↓proliferation	miR-26a targets activators of the cell cycle and Ezh2, a component of PRC2, with repressive functions on negative regulators of the cell cycle	[43]
miR29a	R	CCND2	Cell cycle	↓proliferation	miE29a inhibits proliferation by acting on cyclin D2	[70]
miR-29a; -30a; -141	R	CCNA2–CDK6	Cell cycle	↓proliferation	miR-29a, miR-30a and miR-141 reduce expression of Cyclin A2, reducing cell proliferation	[71]
miR29b-3p	R; Zb	NOTCH2	Notch	↓proliferation	miR29b-3p inhibits CMs proliferation by targeting NOTCH2	[72]
miR-33a-5p	Hu	NOMO1	Nodal	↓proliferation ↑apoptosis ↓differentiation	miR-33a-5p targets NOMO1, a component of a protein complex involved in Nodal signaling leading to apoptosis	[41]
miR-34a	M; R	Bcl2, Sirt1, CCND1	/	↓proliferation ↑apoptosis	miR-34a directly regulated cell cycle and death via modulation of its targets such as Bcl2, Cyclin D1, and Sirt1	[73]
miR-128	R	SUZ12	Cell cycle	↓proliferation	miR-128 downregulates SUZ12, which cannot suppress p27 expression and activate the positive cell cycle regulators Cyclin E and CDK2	[74]
miR-133a	M	CCND2 SRF	Cell cycle Akt	↓proliferation	miR-133 functions as an inhibitor of cardiomyocyte proliferation, targeting cyclin D2 and SRF.	[75]
miR-143-3p	M	YAP–Ctnnd1	Hippo	↑apoptosis	miR-143-3p inhibited the mitosis of CMs, targeting YAP and Ctnnd1	[76]
miR-144	R	TBX1	Jak2/Stat1	↑apoptosis	miR-144 leads to proliferation via TBX1/JAK2/STAT1 axis	[77]
miR-195	M	Chek1	Cell cycle	↓proliferation	miR-195 regulates the expression of many cell cycle genes, including Chek1	[30]
miR-208a	R	PI3K	Akt	↑apoptosis ↑autophagy	miR-208a inhibits the PI3K/AKT pathway	[36]
miR-322; -503	M	IGF2 FGF9	Akt	↓proliferation	They suppress FGF9 and IGF2, disrupting epicardial signaling and impairing myocardial growth	[44]
miR-423	R	MYBL2	WNT	↑apoptosis	miR-423 silences MYBL2 with suppression of wnt/β-	[78]
miR-484	R	YAP1	Hippo	↑apoptosis ↑inflammation	Catenin signaling pathway	[79]
miR-489	R	SPIN1	Akt	↑apoptosis	It directly targets Yap1mRNA to inhibit cell viability	[37]
miR-873	R	GLI1	Hedgehog	↓proliferation	It inhibits SPIN1 thereby inactivating the Akt pathway	[80]

Abbreviations: Hu: human; M: murine; R: rat; Zb: zebrafish.; ↑: upregulation/increase; ↓: downregulation/decrease.

## Data Availability

Not applicable.

## Supplementary Materials

The following supporting information can be downloaded at: https://www.mdpi.com/article/10.3390/ijms241713268/s1. References [215,216,217,218,219,220,221,222,223,224,225,226,227,228,229,230,231,232,233,234,235,236,237,238,239,240,241,242,243,244,245,246,247,248,249,250,251,252,253] are cited in the supplementary materials.

## Abbreviations

ALDH2	Aldehyde Dehydrogenase 2
bFGF	Basic Fibroblast Growth Factor
CCL2	Monocyte Chemoattractant Protein-1 (CCL2/MCP1)
CCR2	CC chemokine Receptor 2
CFs	Cardiac Fibroblasts
Check1	Checkpoint Kinase 1
CMs	Cardiomyocytes
COL1	Collagen Type 1
CTGF	Connective Tissue Growth Factor
CV	Cardiovascular
DAMPs	Damage-Associated Molecular Patterns
DCs	Dendritic Cells
DMRs	Differently Methylated Regions
ECM	Extracellular Matrix
ECs	Endothelial Cells
eNOS	Endothelial Nitric Oxide Synthase
ESCs	Embryonic Stem Cells
FGF9	Fibroblast Growth Factor-9
FPR1	Formyl Peptide Receptor 1
GM-CSF	Granulocyte-Macrophage Colony-Stimulating Factor
HDACs	Histone deacetylases
HIF-1α	Hypoxia-Inducible Factor 1-alpha
I/R	Ischemia/Reperfusion
ICAM-1	Intercellular Adhesion Molecule 1
IGF2	Insulin-like Growth Factor-2
IRA	IL-1 Receptor Antagonist
Klf2	Krupple-like factor 2
lncRNAs	Long Non-coding RNAs
LV	Left Ventricular
MCP-1	Monocyte Chemoattractant Protein-1
MCPs	Mast Cell-Committed Progenitors
MCs	Mast Cells
M-CSF	Macrophage-Colony Stimulating Factor
MCTC	Trypase positive, chymase positive mast cells
MI	Myocardial Infarction
MIP-1α	Macrophage Inflammatory Protein 1 Alpha
miRNAs	MicroRNAs
MMPs	Metalloproteinases
ncRNAs	Non-coding RNAs
NKIRAS2	NF-kB Inhibitor Interacting Ras like 2
NOMO1	Nodal Modulator 1
PDGF	Platelet-Derived Growth Factor
PI3K	Phosphoinositide 3-kinase
PRR	Pathogen Recognition Receptors
ROS	Reactive Oxygen Species
SIRT	Sirtuin
SOCS3	Suppressor Of Cytokine Signaling 3
SPARC	Secreted Protein Acidic and Rich in Cysteine
SPIN1	Spindlin-1
TGF-β	Transforming Growth Factor-beta
TIMP-1	Tissue metalloproteinase inhibitor-1
TLRs	Toll-Like Receptors
TNF-α	Tumor Necrosis Factor-alpha
Treg	Regulatory T cells
TSP-1	Thrombospondin-1
TSP-2	Thrombospondin-2
VCAM-1	Vascular Cell Adhesion Molecule 1
VEGF	Vascular-Endothelial Growth Factor
VLA-4	Very Late Antigen-4
VSMCs	Vascular Smooth Muscle Cells
WAT	White Adipose Tissue
