# Supplementary material for: miRNAs Epigenetic Tuning of Wall Remodeling in the Early Phase after Myocardial Infarction: A Novel Epidrug Approach
Source: Int J Mol Sci. 2023 Aug 26;24(17):13268. doi: 10.3390/ijms241713268 (PMC10487654; doi:10.3390/ijms241713268)
Supplement: Supplementary file 1 [file ijms-24-13268-s001.zip › ijms-2568325-supplementary.pdf]

Table S1. Summary of the effects of miRNAs during the inflammatory phase

miRNA	Model	Targets	Mechanism(s)	Ref.
**CMs apoptosis**				
miR-15 family	P	Arl2, Bcl2	miR-15 family influences cardiomyocyte cell survival by regulating the expression of many pro-survival proteins, such as Arl2 and Bcl2	[215]
miR-24	M	/	miR-24 could reduce cardiomyocytes apoptosis post-ischemia	[216]
miR-26b	R	GATA4	miR-26b targets GATA4, reducing endothelin-induced CM hypertrophy and sensitizes the cells to apoptotic insults	[217]
miR-34a	R	ALDH2	miR-34a stimulates cardiomyocytes apoptosis, by down-regulating ALDH2	[136]
miR-92a	R	Smad7	miR-92a inhibits Smad7, a transcription factor that protects against apoptosis by negative regulating of NF-kB	[218]
miR-101	M	DDIT4	miR-101 reduces autophagy and apoptosis of cardiomyocytes, attenuating MI-induced injury	[139]
miR-101	R	RUNX1	miR-101 suppresses cardiomyocyte apoptosis after MI, by downregulating RUNX1	[138]
miR-122	R	GATA-4	miR-122 regulates apoptosis in CMs, by targeting GATA-1, a transcription factor involved in CM proliferation/survival	[219]
miR-124	M	Dhcr24	miR-124 promotes cardiomyocytes apoptosis, directly targeting Dhrc24, involved in anti-apoptotic processes	[135]
miR-130a-3p	Hu	PDE4D	miR-130a-3p reduces aberrant cardiomyocytes apopotosis, via targeting PDE4D	[220]
miR-132	R	IL-1β	miR-132 protects against cardiomyocytes apoptosis and myocardial remodeling, by targeting IL-1β	[221]
miR-133a	M	Bim, Bmf	miR-133 promotes cardiac progenitor cells survival and improves their capacity to protect the heart against hypertrophy and apoptosis	[134]
miR-144-3p	M	/	Overexpression of miR-144-3p reduces the cardiomyocyte apoptosis	[222]
miR-145	M, R	Bnip3	miR-145 protects against the activation of endogenous apoptotic pathway in cardiomyocytes under oxidative stress by targeting Bnip3	[223]
miR-429	Hu	Notch1	miR-429 inhibits Notch1 expression and therefore its anti-apoptotic action versus cardiomyocytes. The downregulation of miR-429 enhances CM viability and proliferation	[137]
**Inflammation**				
miR-19a/b	M	/	Up-regulation of some immune response genes, such as Arg-1 and CD162	[34]
miR-21		KBTBD7, NF-kB	Mir-21 inhibits KBTBD7-mediated promotion of NF-kB signaling pathway, ameliorating inflammatory response	[224]
miR-22	M; Zb	VE-cadherin	miR-22 induces endothelial cell and tissue inflammation, and causes abnormal angiogenesis, blocking the expression of VE-cadherin	[225]
miR-29a	M; R	SIRT1	miR-29a inhibits SIRT1, blocking its anti-oxidative and cardioprotective effects and stimulating pyroptosis	[140]
miR-132	M	SIRT1	miR-132 inhibits SIRT1 and PGC-1α/Nrf2 signaling, with subsequent upregulation of MDA and iNOS and repression of SOD and eNOS, contributing to oxidative stress	[141]
miR-155	M	SIRT1	miR-155 targets SIRT1, inhibiting its protective effects on myocardial	[142]
miR-208a	R	CHD9	Mir-208a reduces inflammation and oxidative stress inhibiting CHD9, an activator of Notch pathway	[226]

Fibrosis				
miR-1	R	Cyclin D2/CDK6	In cardiac fibroblasts, miR-1 targets Cyclin D2 and CDK6, blocking basal and TGFβ-induced CF proliferation. Unfortunately, after MI, miR-1 is downregulated by Jak2/STAT3 signaling pathway	[227]
miR-15 family	R, Hu	TGFβ signaling	miR-15 family targets both activators (TGFβR, SMAD3, endoglin) and repressor (SMAD7) of TGFβ signaling pathway, regulating both cardiac fibrosis and cardiomyocytes hypertrophy	[228]
miR-19b	R	PTEN	miRNA promotes cardiac fibroblasts proliferation and migration	[229]
miR-21	M	Smad7	miR-21 is upregulated after MI by TGFβ1, with subsequent reduction of Smad7 expression, increased phosphorylation of Smad2/3 and promoted CFs activation	[230]
miR-21	R	CADM1	miR-21 suppresses CADM1, resulting in upregulation of STAT3 expression and CF proliferation	[231]
miR-21	R	Jagged1	miR-21 mediates TGFβ1 induced cardiac fibroblast-to-myofibroblast switch and myocardial fibrosis by targeting Jagged1 and blocking the Notch signaling pathway	[232]
miR-92a	M	Smad7	miR-92a is up-regulated in cardiomyocytes after MI and è transfers to cardiac fibroblasts within exosome, where reduces Smad7-mediated inhibition of αSMA transcription and stimulates the conversion to myofibroblasts	[147]
miR-125b	Hu, M	P53/apelin	miR-125b suppresses apelin and p53, inducing CF proliferation and fibroblast-to-myofibroblast switch	[233]
miR-130a	M	TGFβR	miR-130a negatively regulates cardiac fibrosis after MI, by targeting TGFβR1 and decreasing the activity of TGFβ/Smad signaling, to inhibits the transformation of CFs in myofibroblasts	[143]
miR-133a	R	TGFβ1, CTGF	miR-133a reduces myocardial infarction area, the synthesis of extracellular matrix collagen and the differentiation of cardiac fibroblast into myofibroblasts, by decreasing TGFβ1 and CTGF expression	[144]
miR-144-3p	P	PTEN	miR-144-3p promotes cardiac fibroblasts proliferation, migration, and collagen production by targeting PTEN	[234]
miR-146b-5p	M; Mp	IRAK1, CEACAM1	miR-146b-5p activates fibroblast proliferation and migration, and fibroblast-to-myofibroblast transition, impairs endothelial cell function and stress survival, and disturbs macrophage paracrine signaling, targeting interleukin 1 receptor associated kinase 1 (IRAK1) and carcinoembryonic antigen related cell adhesion molecule 1 (CEACAM1)	[235]
miR-148b	R	/	miR-148b knockdown the myofibroblast conversion, proliferation, and collagen production	[145]
miR-155	M	/	miR-155 appears to be involved in the differentiation of cardiac fibroblasts into myofibroblasts	[236]
miR-195	M	SMAD7	miR-195 is secreted by damaged cardiomyocytes within cardiosomes and transferred to cardiac fibroblasts, where it reduces SMAD7-mediated inhibition of αSMA transcription, leading to myofibroblast conversion	[148]
miR-214	R	Mfn2	miR-214 can mediate proliferation and collagen synthesis in cardiac fibroblasts by regulating Mfn2 and its downstream ERK1/2 signalling pathway	[237]
miR-223	R	RASA1	miR-223 is a potent pro-fibrotic factor. It reduces the expression of RASA1, inducing CFs proliferation, migration, and differentiation	[238]
miR-590-3p	P, H	ZEB1	miR-590-3p suppresses proliferation, differentiation, migration, and collagen synthesis of cardiac fibroblasts, significantly reducing mRNA levels of α-SMA, Col1A1 and Col3A1, by targeting the transcription factor ZEB1	[146]

Abbreviations: Hu: human; M: murine; P: pig; Mp: minipig; R: rat; Zb: zebrafish.

Tabel S2. Summary of the effects of miRNAs during the proliferative phase

miRNA	Model	Targets	Mechanism(s)	Ref.
**Angiogenesis**				
miR-27a	M	VE-cadherin	miR-27a inhibits VE-cadherin, blocking capillary tube formation and angiogenesis	[215]
miR-92a	R	KLF4/MKK4	miR-92a regulates KLF4 and MKK4 expression in endothelial cells, reducing endothelial proliferation and migration and increasing neointimal hyperplasia	[239]
miR-126	Hu	HIF-1α	miR-126 modulate HIF-1α protein levels, regulating angiogenesis, vascular integrity, and vessel maturation	[172]
miR-129-1, miR-133	Hu	VEGFR2, FGFR1	miR-129-1 and miR133 reduce proliferation rate, cell viability and migration activity of human umbilical vein endothelial cells (HUVEC), targeting VEGFR2 and FGFR1.	[173]
miR-130a	M; Hu	PTEN	Suppression of PTEN with activation of PI3K/Akt signalling, promoting endothelial cell proliferation/migration	[177]
miR-133a	R; M	GCH1	miR-133a downregulates GCH1 in endothelial cells, resulting in endothelial dysfunction	[240]
miR-139-5p	Hu	VEGFR-1	miR-139-5p inhibits the viability of endothelial cells, targeting VEGFR-1	[174]
miR-199b	M	JAG1	miR-199b targets the Notch ligand JAG1, resulting into VEGF transcriptional activation and *via* STAT3, with subsequent stem cell differentiation to endothelial cells and angiogenesis	[175]
miR-200a-3p	R; M	PIGF/VEGF-A	miR-200a-3q alters the angiogenic potential, proliferation, and migration of endothelial cells, increases their apoptosis and induces their dysfunction, by modulating PIGF-dependent VEGF-A signaling	[176]
miR-208	/	PPAR, ACTA2, ROR2, PI3K/Akt	miR-208 promotes dysfunction of blood vessels, by targeting proteins associated with inflammation, endothelial apoptosis, vascular smooth muscular cells proliferation and migration	[178]
miR-375	M; Hu	PDK-1/Akt	Inhibition of miR-375 promotes neovascularization, reduces cardiomyocyte apoptosis, mediates M1 to M2 macrophage switch, limiting the infarct size and the LV dysfunction	[188]
**Fibrosis**				
miR-24	M	Furin	miR-24 limits the expression of furin, a protease which regulates angiotensin II-induced TGFβ activation, improving heart function by regulating cardiac fibrosis	[216]
miR-26a	R	Col1, CTGF	miR-26a regulates cardiac fibrosis, by directly targeting collagen I and CTGF	[179]
miR-29b	R	Col1a1, Col3a1, α-SMA	miR-29b inhibits fibrosis-related genes expression, but usually it is in turn blocked by ROS-mediated Smad3 activation	[182]
miR-29b	R	SH2B3	miR-29b alleviates cardiac fibrosis and dysfunction by targeting SH2B3 and thus down-regulating the expression of COL1A1 and αSMA	[180]
miR-34 family	M	VEGF, vinculin, POFUT1, Notch1, semaphorin4B	miR-34 family is involved in pathological cardiac remodeling. Its inhibition improves heart function, reducing cardiac fibrosis and increasing angiogenesis, among the others	[241]

miR-34a	M	/	miR-34a induces cardiac fibrosis-associated collagens and other cellular matrix production through the induction of the TGFβ1/Smad4 signaling pathway	[242]
miR-208	/	GATA4	miR-208 inhibits the expression of COL1 and ACTA2, by downregulating GATA4, hence hampering the progression of post-infarction cardiac fibrosis	[178]
miR-208a	R	CHD9	miR-208a permits the expression of genes involved in cardiac hypertrophy and fibrosis, by targeting CHD9 and inhibiting Notch signaling pathway	[181]
miR-378	M; R	GRB2, KSR1, MAPK1, MKK6	miR-378 plays a dual role in inhibiting cardiac hypertrophy, via targeting GRB2, KSR1 and MAPK1, and cardiac fibrosis, by suppressing MKK6 and consequently p38 MAPK and Smad2/3 signaling pathways	[243]
**Macrophage polarization**				
miR-21-5p	M	TLR4	Exosomal miR-21-5p from MSC may be involved in the macrophages polarization to M2 phenotype, and in alleviating the inflammatory response, plausibly by targeting the TLR4/PI3K/Akt signaling pathway	[183]
miR-24-3p	Hu	Plcb3	Exosomal miR-24-3p from human umbilical cord mesenchymal stem cells inhibits Plcb3 expression and the subsequent NF-kB pathway activation, promoting M2 macrophages polarization	[186]
miR-27a-5p	M	Ppm1l	miR-27a-5p downregulates Ppm1L expression, inhibiting M2-like macrophages polarization	[244]
miR-146a-5p	M	/	Exosomal miR-146a-5p from cardiomyocytes induces M1 polarized macrophages	[245]
miR-150	M	STAT1	miR-150 downregulates STAT1, promoting the expression of M2 polarization marker genes	[184]
miR-182	M	TLR4	miR-182 reduces inflammation by negatively polarizing M1 macrophages, va downregulation of TLR4/NF-kB pathway	[185]
**Cardiomyocyte proliferation**				
miR-19a/b	M	PTEN	miR-19a/19b blocks PTEN expression by inducing CMs proliferation mediated by the AKt signaling pathway	[34]
miR-19b-3p	Hu; Zb	Notch2	Mir-29b-3p inhibits cardiomyocyte proliferation	[72]
miR-106a-363 cluster	/	Notch3	This miR cluster stimulates cell cycle re-entry of cardiomyocytes by repressing the Notch3 pathway	[187]

Abbreviations: Hu: human; M: murine; R: rat; Zb: zebrafish.

Table S3. Summary of the effects of miRNAs during the maturation phase

**Cardiac remodelling**				
**miRNA**	**Model**	**Targets**	**Mechanism(s)**	**Ref.**
miR-22	M; Hu	EVI1	Cardiac-specific overexpression of miR-22 causes a pro-hypertrophic gene expression profile resulting in the generation of cardiac dilatation and heart failure	[246]
miR-101	R	RUNX 1	miR-101 blocks the TGF-β1/Smad2 signaling pathway, and thus protects heart against MI-induced cardiac remodeling, by inhibiting RUNX1	[138]
miR-122	R	FoxO3	miR-122 blocks FoxO3, promoting calcineurin signaling pathway activation and accelerating CM hypertrophy	[247]
miR-132	R	PTEN	miR-132 helps to improve cardiac dysfunction and impairment of cardiac hemodynamics, and reduces cardiac fibrosis in heart failure, by inhibition of PTEN	[248]
miR-145	R	KLF5	miR-145 is involved in VSMCs differentiation. Its reduction increases the expression of KLF5 which in turn decreases VSMC differentiation marker genes via its downstream molecule, myocardin	[249]
miR-155	M	Jarid2	miR-155 inhibits Jarid2, which in turn represses the expression of ANF, a hallmark of cardiac hypertrophy	[250]
miR-181c	M; R	mt-COX1	miR-181c enhances heart damage via overproduction of ROS via targeting mt-COX1 in the myocytes mitochondrial	[251]
miR-212/132 family	M; R	FoxO3	miR-212/132 family down-regulates FoxO3, with activation of the pro-hypertrophic calcineurin/NFAT pathway	[252]
miR-486	R	SRSF3	miR-486 improves fibrotic activity, pathological remodelling, and scar size, by targeting SRSF3, with subsequent increase in p21 signaling, which results in cellular senescence of myofibroblasts	[253]

Abbreviations: Hu: human; M: murine; R: rat.
